# Whole Exome Sequencing in Females with Autism Implicates Novel and Candidate Genes

**DOI:** 10.3390/ijms16011312

**Published:** 2015-01-07

**Authors:** Merlin G. Butler, Syed K. Rafi, Waheeda Hossain, Dietrich A. Stephan, Ann M. Manzardo

**Affiliations:** 1 Departments of Psychiatry & Behavioral Sciences, University of Kansas Medical Center, 3901 Rainbow Boulevard, MS 4015, Kansas City, KS 66160, USA; E-Mails: rafigene@yahoo.com (S.K.R.); whossain@kumc.edu (W.H.); amanzardo@kumc.edu (A.M.M.); 2 Department of Human Genetics, University of Pittsburgh, Pittsburgh, PA 15260, USA; E-Mail: dstephan@pitt.edu

**Keywords:** whole exome sequencing, females, autism spectrum disorder, genetic variants, X chromosome inactivation

## Abstract

Classical autism or autistic disorder belongs to a group of genetically heterogeneous conditions known as Autism Spectrum Disorders (ASD). Heritability is estimated as high as 90% for ASD with a recently reported compilation of 629 clinically relevant candidate and known genes. We chose to undertake a descriptive next generation whole exome sequencing case study of 30 well-characterized Caucasian females with autism (average age, 7.7 ± 2.6 years; age range, 5 to 16 years) from multiplex families. Genomic DNA was used for whole exome sequencing via paired-end next generation sequencing approach and X chromosome inactivation status. The list of putative disease causing genes was developed from primary selection criteria using machine learning-derived classification score and other predictive parameters (GERP2, PolyPhen2, and SIFT). We narrowed the variant list to 10 to 20 genes and screened for biological significance including neural development, function and known neurological disorders. Seventy-eight genes identified met selection criteria ranging from 1 to 9 filtered variants per female. Five females presented with functional variants of X-linked genes (*IL1RAPL1*, *PIR*, *GABRQ*, *GPRASP2*, *SYTL4*) with cadherin, protocadherin and ankyrin repeat gene families most commonly altered (e.g., *CDH6*, *FAT2*, *PCDH8*, *CTNNA3*, *ANKRD11*). Other genes related to neurogenesis and neuronal migration (e.g., *SEMA3F*, *MIDN*), were also identified.

## 1. Introduction

Classical autism or autistic disorder belongs to a group of genetically heterogeneous conditions known as Autism Spectrum Disorders (ASD) characterized by three clinical features: (1) marked impairments in verbal and non-verbal communication; (2) impairments in social interactions; and (3) restricted repetitive behaviors and interests [1,2]. Additional findings include lack of eye contact, tactile defensiveness and sleep disturbances with diagnosis validation at a young age requiring test instruments such as Autism Diagnostic Observation Schedule (ADOS) and Autism Diagnostic Interview-Revised (ADI-R) [3,4,5]. Latest estimates reveal that ASD affects about 1 of every 68 children by 8 years of age with a predominant male to female (4:1) ratio [6]. This occurrence rate is higher than that of epilepsy or Down syndrome. Congenital anomalies, intellectual disability, growth retardation and/or seizures can occur in a subset of children presenting with features related to autism. Regression of skills is observed in about 30% of cases with ASD and about 60% show intellectual disabilities [7,8]. Small or large head size is seen in about 30% of affected children with ASD with extreme macrocephaly associated with mutation of the *PTEN* tumor suppressor gene [9]. Heritability studies to identify the contribution of genetic factors in autism have shown an estimate as high as 90%. Recognized single gene conditions such as fragile X syndrome, Rett syndrome or tuberous sclerosis account for less than 20% of all cases with ASD [10].

Standard routine chromosome studies in individuals with ASD have shown abnormalities of over one dozen chromosomes. Various cytogenetic findings are reported including deletions, duplications, translocations and inversions often involving the chromosome 15q11-q13 region or the 22q11.2 band [10,11]. More advanced chromosome microarray studies are more powerful in finding cytogenetic abnormalities than routine chromosome studies. High resolution microarrays have detected recurrent small submicroscopic deletions or duplications in individuals with ASD indicating the presence of hundreds of candidate and/or known ASD genes localized to each human chromosome. In addition, there are numerous submicroscopic copy number changes, more often of the deletion type, seen in greater than 20% of patients with ASD using microarray analysis [11]. Many of these chromosome abnormalities contain genes playing a causative role in ASD. In a recent review of genetic linkage data, candidate genes and genome-wide association studies along with further advances in genetic technology including high resolution DNA microarray and next generation sequencing have led to a compilation of 629 clinically relevant candidate and known genes for ASD [12].

Given the fact that females with ASD are historically understudied, we performed whole exome sequencing of well-characterized females with classical autism from multiplex families in the search for existing or potentially new candidate genes for autism. We utilized a cohort of affected females recruited by the Autism Genetic Research Exchange (AGRE), a gene bank housing data and biospecimens from over 2000 families (www.AGRE.autismspeaks.org). Most families had two or more affected children with autism. Identification of causative mutations (e.g., serotonin-related gene mutations and disturbed biology) could be important to guide selection of treatment options and medication use as well as to manage medical co-morbidities such as seizures, developmental regression (e.g., *MECP2* gene) or for cancer (e.g., *PTEN* gene). A cursory autism data base search revealed a large body of publications, particularly since 2008, linking autism to a wide range of genetic and environmental factors found only 3 (0.48%) clinically relevant ASD genes to be located on the Y chromosome while 68 (10.81%) clinically relevant ASD genes were recognized on the X-chromosome [12].

The preponderance of males with ASD may be attributable to the single X chromosome in males depriving the normal allelic pair of genes due to the XY sex chromosome constitution. Hence, sex chromosomes illustrate the most obvious genetic difference between men and women. All female mammals have two X chromosomes and achieve a balanced X chromosome gene expression with males by inactivating one of their X chromosomes, a process known as X chromosome inactivation (XCI) [13]. This process occurs randomly and very early in embryonic development. Once an X chromosome is “selected” for inactivation within a cell, then the same X chromosome remains inactivated in each subsequent daughter cell. Therefore, females have a mixture of cells with random expression of genes on a single X chromosome. Occasionally, XCI represents a nonrandom pattern or high skewness which is usually defined by at least 80% preferential inactivation of one of the two X chromosomes [14,15]. Skewed XCI appears to play a role in the increased incidence and presentation of diseases in females with known X-linked gene involvement such as Rett syndrome [16], X-linked intellectual disability [14], X-linked adrenoleukodystrophy [17] and possibly autism [15]. Skewed XCI may also reflect an early disruption in the developing embryo causing cell death followed by a small number of dividing cells repopulating the embryo [18,19,20]. XCI can be measured by using the androgen receptor (*AR*) gene located at chromosome Xq13 which contains a highly polymorphic CAG repeat region and normally inactivated on one of the X chromosomes in females. The *AR* gene is used most often to determine the XCI status [21,22]. Disorders with an imbalance in the number of males versus females affected as in autism become a likely condition to study XCI and its impact on X-linked genes in females. We suspect additional autosomal and/or sex chromosome based ASD gene(s) that either overwhelms the protective normal alleles of the second X chromosome among females, or preferentially silences them due to a plausible skewed X chromosome inactivation among neural cell lineages. Therefore, the whole exome sequence of females with ASD from multiplex families having more than one family member affected was analyzed in our study with next generation sequencing and bioinformatics with a novel machine-learning classification engine applied to identify the contributing ASD genes and the proportion of disease contributing alleles from the X chromosomes versus autosomes. The inactivation status of the two X chromosomes was determined to identify significant deviation from randomness, taking into consideration whether the X chromosome gene is known to escape inactivation, or known to act as a dominant gene.

## 2. Results

Whole exome sequencing from DNA of 30 females with autism spectrum disorders from AGRE identified between 100 and 300 genes showing genomic variants of novel or candidate genes for autism per subject with an initial classification score >0.5. To further narrow the list of putative disease causing genes, we increased the level of selection criteria by adding cutoff levels for other predictive parameters (GERP2, PolyPhen2 and SIFT) and specifically increased the classification score from >0.5 to a more stringent score of >0.7. For example, the initial list of genes and genomic variants for Subject HI2898 consisted of 245 genes when using a classification score >0.5 alone and then reduced to 22 genes when the cutoff levels of other predictive parameters (GERP2, PolyPhen2 and SIFT) were included at the chosen final selection criteria level. Finally, the classification score was raised to >0.7 to generate the master list of 14 genes. For the 30 females with autism, the number of genes identified based on the final selection criteria ranged between 8 to 23 genes with 10 females presenting with an X-linked gene in the list of selected genes meeting the final selection criteria. The putative candidate genes and genomic variants were then subjected to further screening for biological significance and relevance for neural development, function and for causation of known neurological disorders based on published medical literature. In so doing, we identified two potentially causative genes for autism for Subject HI2898. See Table 1 for the list of genes meeting or exceeding the established final selection criteria level and screening for biological significance. Identical genomic variants were recognized for several subjects even after increasing the final level of selection criteria required to reduce the number of candidate genes and considered artifactual findings. These genes with identical genomic variants included *KCNJ12*, *MLL3*, *OR9G1* and *PCMTD1* with different mutations reported in other disease states and appeared to reflect artifacts in the present analysis of autism.

We report genomic coordinates, previously recognized SNPs (and their ID numbers), reference base to alternate base sequences (e.g., G→A), variant call format (VCF) information, SNP effects (synonymous vs non- synonymous), SNP codons, and GERP2, PolyPhen2, SIFT, Blosum, Blosum62 and the classification scores for each genomic variant identified per affected female (Table 1). Our analysis identified a total of 79 different genes containing variants (both known and novel) consisting of 12 (15%) known candidate genes for ASD, 24 (30%) paralogues of known candidate ASD genes and 43 (54%) novel genes with functional roles in nerve cell growth, adhesion and neurodevelopment or function as putative candidate ASD genes (See Table 2). The identified candidate ASD genes and paralogues were distributed across 11 (37%) and 16 (53%) of the females with autism, respectively. 

Four females (HI0555, HI0890, HI1402 and HI2126) possessed a single variant of significance in clinically relevant candidate or known ASD genes considered conclusive for causality. Seventy-seven percent of subjects (*N* = 23) possessed multiple variants with possible influences on causation of ASD. Two (9%) of the 23 females (7% of the total sample population) possessed more than five variants/mutations of known candidate ASD genes, paralogues or other functionally relevant genes with presumed influence over the development of ASD. Three (10%) of the total females (HI1143, HI1884 and HI2278) possessed a single gene variant with possible functional relevance toward the development of ASD but no known candidate ASD genes or paralogues identified to be considered conclusive.

**Table 1:** Putative disease causing genes for autism identified using whole exome sequencing of females with autism spectrum disorder (ASD).

ID Number XCI	Gene Symbol (Category) *	Chromosome Position (Hg19)	Genomic Variant					Classification Score	GERP2	PolyPhen2 Score	SIFT Score	Blosum Score/Blosum62
			SNP Effect	Amino Acid	SNP Codon	SNP Function	VCF Allele Depth					
**HI0405 46%:54%**	*KCNC2* (P) [12]	12:75444895	Non-synon	p.F296C	tTt/tGt	Missense	162, 30	0.790	0	0.984	0	0.787/−2
	*ASPM* (F) [23]	1:197112823	Non-synon	p.R186G	Aga/Gga	Missense	69, 53	0.777	0.007	0.994	0	0.787/−2
	*TAF3* (F) [24]	10:8006662	Non-synon	p.R396G	Cga/Gga	Missense	69, 60	0.740	0	0.956	0	0.787/−2
	*FLRT2* (F) [25]	14:86089552	Non-synon	p.H564R	cAt/cGt	Missense	28, 29	0.736	0	0.958	0.02	0.787/0
	*SYTL4* (F) [26]	X:99941091	Non-synon	p.H448D	Cat/Gat	Missense	36, 37	0.710	0.006	1	0.03	0.787/−1
**HI0555/58%:42%**	*SETD2* (A) [12,27,28]	3:47164293	Non-synon	p.K610O	aaG/aaT	Missense	23, 21	0.778	0	0.970	0	8.522/0
**HI0558 52%:48%**	*IFT122* (F) [29]	3:129238526	Non-synon	p.R986H	cGc/cAc	Missense	58, 61	0.795	0.008	0.989	0	0.074/0
	*NUP98* (F) [30]	11:3756456	Non-synon	p.R519W	Cgg/Tgg	Missense	12, 9	0.793	0.006	0.999	0	24.449/−3
	*ZKSCAN1* (F) [31]	7:99621487	Non-synon	p.R119C	Cgc/Tgc	Missense	8, 5	0.782	0.006	1	0	0.787/−3
	*DNAH8* (F) [32]	6:38834433	Non-synon	p.M1971V	Atg/Gtg	Missense	8, 14	0.783	0.009	0.902	0	4.182/1
	*CENPJ* (F) [33]	13:25487103	Non-synon	p.M20L	Atg/Ttg	Missense	45, 42	0.761	0.007	0.991	0.01	4.312/2
	*TRIM5* (F) [34]	11:5878550	Non-synon	p.R127P	cGc/cCc	Missense	42, 24	0.742	0.0089	0.971	0	0.787/−2
	*PCDH8* (A) [35]	13:53421358	Non-synon	p.A404V	gCg/gTg	Missense	13, 14	0.731	0	0.924	0	0.787/0
	*CTNNA3* (A) [36]	10:68381521	Non-synon	p.M434V	Atg/Gtg	Missense	30, 17	0.723	0.009	0.942	0.01	4.183/1
	*PPP1R18* (P) [37]	6:30652604	Non-synon	p.S397P	Tct/Cct	Missense	12, 6	0.703	0.005	0.958	0	0.787/−1
**HI0605 53%:47%**	*CCDC64* (A) [38]	12:120428012	Non-synon	p.A113T	Gcc/Acc	Missense	13, 24	0.794	0.006	0.96	0	4.774/−1
	*DCHS1* (F) [39]	11:6662745	Codon Change Plus Codon Insertion	p.L32LW	ctg/ctCTGg	None	11, 8	0.700	0	0.979	0.02	0/0
**HI0714 40%:60%**	*TRAK2* (F) [40]	2:202264189	Non-synon	p.R130G	Cga/Gga	Missense	31, 27	0.805	0.010	1	0	0.064/−2
	*MPST* (F) [41]	22:37425405	Utr_3_Prime	p.Null-1Null	Null	None	20, 21	0.731	0.008	1	0	0.787/−2
	*FSTL3* (F) [42]	19:680331	Non-synon	p.R115H	cGc/cAc	Missense	7, 3	0.729	0.010	1	0.01	8.723/0
**HI0751 16%:84%**	*IST1* (F) [43,44]	16:71956522	Non-synon	p.P232R	cCc/cGc	Missense	63, 50	0.754	0.008	0.972	0.01	0.787/−2
	*KIF27* (P) [43,44]	9:86474259	Non-synon	p.S921P	Tca/Cca	Missense	22, 25	0.730	0.009	0.970	0.01	0.787/−1
	*PCDHGA1* (P) [45]	5:140870234	Non-synon	p.V475A	gTg/gCg	Missense	152, 141	0.713	0	0.995	0	0.787/0
**HI0765 77%:23%**	*SEMA3F* (P) [46]	3:50225525	Non-synon	p.R679W	Cgg/Tgg	Missense	21, 7	0.793	0.006	1	0	0.787/−3
	*SCN11A* (P) [47]	3:38968409	Frame-shift	p.-166SS?	-/TCTT CACT	None	23, 17	0.748	0.008	0.982	0	0/0
	*GPRASP2* (F) [48]	X:101972234	Non-synon	p.R812C	Cgt/Tgt	Missense	69, 51	0.706	0	0.989	0.01	0.787/−3
**HI0793 37%:64%**	*ELTD1* (F) [49]	1:79356850	Non-synon	p.C687R	Tgt/Cgt	Missense	50, 25	0.807	0.008	0.998	0	10.199/−3
	*DENND3* (F) [50]	8:142161854	Non-synon	p.S252L	tCg/tTg	Missense	40, 20	0.806	0.006	0.992	0	0.787/−2
	*MIDN* (F) [51]	19:1250353	Non-synon	p.C19G	Tgc/Ggc	Missense	8, 3	0.793	0.008	0.998	0	17.850/−3
	*RADIL* (F) [52]	7:4917586	Non-synon	p.P61R	cCt/cGt	Missense	42, 27	0.774	0.004	1	0	14.302/−2
	*EXOC7* (P) [53,54]	17:74097856	Non-synon	p.R71Q	cGg/cAg	Missense	36, 46	0.755	0.006	0.992	0.01	0.059/1
	*PLCE1* (F) [55]	10:96064250	Non-synon	p.H1515Y	Cat/Tat	Missense	22, 14	0.737	0.008	0.999	0.02	7.078/2
	*KDM5A* (F) [56]	12:416817	Non-synon	p.A1244T	Gcc/Acc	Missense	42, 25	0.706	0	0.993	0	0.157/0
	*ARAP2* (F) [57]	4:36115873	Non-synon	p.D1358N	Gat/Aat	Missense	70, 53	0.702	0.008	0.907	0.02	12.052/1
**HI0855 44%:56%**	*CHAC1* (F) [58]	15:41247844	Frame- shift	p.-177?	-/T	None	23, 19	0.941	0.009	0.997	0	0.787/0
	*MIDN* (F) [59]	19:1250353	Non-synon	p.C19G	Tgc/Ggc	Missense	3, 7	0.793	0.008	0.998	0	17.85/−3
	*CDH6* (P) [60]	5:31323179	Non-synon	p.R712G	Aga/Gga	Missense	76, 81	0.749	0.003	0.988	0.01	0.787/−2
**HI0868 58%:42%**	*CHST3* (P) [61]	10:73767391	Non-synon	p.Y200C	tAc/tGc	Missense	43, 30	0.759	0	0.998	0.02	17.489/−2
	*FBXO5* (P) [62]	6:153296090	Non-synon	p.H256R	cAt/cGt	Missense	103, 76	0.756	0.003	1	0	0.787/0
**HI0890/NI**	*BTAF1* (A) [63]	10:93719892	Non-synon	p.Y414F	tAt/tTt	Missense	29, 16	0.765	0.005	0.997	0	3/3
**HI1143/14%:86%**	*CLPTM1* (F) [64]	19:45476442	Non-synon	p.N94I	aAc/aTc	Missense	30, 27	0.744	0.007	1	0	0.787/−3
**HI1157 61%:39%**	*AMIGO1* (F) [65]	1:110050220	Non-synon	p.R438W	Cgg/Tgg	Missense	42, 28	0.806	0	0.994	0	0.787/−3
	*PEX5* (P) [66]	12:7362354	Non-synon	p.R560C	Cgc/Tgc	Missense	71, 50	0.801	0.006	1	0	0.787/−3
	*KIF23* (P) [67]	15:69718474	Non-synon	p.E266A	gAa/gCa	Missense	52, 48	0.775	0.007	0.998	0	0.787/−1
**HI1228 42%:58%**	*FOXA3* (P) [68]	19:46376266	Non-synon	p.G334R	Gga/Aga	Missense	30, 29	0.801	0.004	0.975	0	26.000/−2
	*FRMD4A* (F) [69]	10:13698719	Non-synon	p.S941L	tCg/tTg	Missense	6, 8	0.775	0	0.957	0	0.064/−2
**HI1305 73%:27%**	*PIR* (F) [70]	X:15474053	Non-synon	p.E132G	gAa/gGa	Missense	32, 28	0.811	0.007	0.962	0	0.213/−2
	*DNAH2* (F) [71]	17:7637815	Non-synon	p.I255T	aTa/aCa	Missense	25, 20	0.793	0.010	0.942	0	7.006/−1
**HI1375 17%:83%**	*LHX2* (F) [72]	9:126777468	Non-synon	p.A130P	Gct/Cct	Missense	50, 34	0.775	0.006	0.998	0	0.787/−1
	*EPHA4* (P) [73]	2:222428829	Non-synon	p.A148T	Gct/Act	Missense	18, 25	0.744	0	0.977	0	0.787/0
**HI1402/85%:15%**	*IL1RAPL1* (A) [12,74]	X:29973282	Non-synon	p.P478Q	cCa/cAa	Missense	57, 42	0.761	0	0.999	0	7.684/−1
**HI1422 56%:44%**	*RTN4R* (F) [75]	22:20230387	Non-synon	p.S89L	tCg/tTg	Missense	11, 18	0.778	0.004	0.998	0	0.064/−4
	*DHX8* (F) [76]	17:41606990	Non-synon	p.R282H	cGc/cAc	Missense	4, 8	0.724	0.010	1	0	0.787/0
**HI1433 93%:7%**	*FAT2* (P) [77,78,79]	5:150947258	Non-synon	p.T411I	aCt/aTt	Missense	32, 29	0.757	0	0.992	0	6.276/−1
	*FITM2* (F) [80,81]	20:42939631	Non-synon	p.R52H	cGc/cAc	Missense	6, 5	0.750	0.008	0.999	0.02	8.723/0
	*RALGPS2* (F) [82,83]	1:179064186	Non-synon	p.V342M	Gtg/Atg	Missense	69, 73	0.734	0.010	0.970	0	0.787/1
**HI1739 31%:69%**	*ASB3* (F) [84]	2:53941656	Non-synon	p.S208N	aGc/aAc	Missense	53, 30	0.773	0.007	0.980	0	0.403/1
	*AKAP6* (F) [85]	14:33291533	Non-synon	p.D1504P	gAt/gTt	Missense	43, 41	0.761	0	0.983	0.01	0.787/−3
	*GRM4* (A) [86]	6:34100940	Non-synon	p.D111N	Gac/Aac	Missense	58, 51	0.726	0.005	0.993	0.02	0.787/1
**HI1884/48%:52%**	*TRAF3IP1* (F) [87]	2:239306221	Non-synon	p.M537T	aTg/aCg	Missense	13, 19	0.759	0.007	0.999	0.01	0.033/−1
**HI1954 23%:77%**	*DDX5* (P) [88]	17:62500102	Non-synon	p.S146C	tCt/tGt	Missense	88, 91	0.788	0.007	0.950	0	0.787/−1
	*KIF5A* (P) [89]	12:57974875	Non-synon	p.R891Q	cGg/cAg	Missense	25, 19	0.767	0.005	0.989	0	0.787/1
**HI2126/41%:59%**	*ANKRD11* (A) [90,91]	16:89351565	Non-synon	p.T461R	aCa/aGa	Missense	20, 11	0.767	0	0.961	0	0.787/−1
**HI2172 61%:39%**	*TNFRSF21* (F) [92]	6:47221105	Non-synon	p.W465R	Tgg/Agg	Missense	8, 16	0.805	0.004	1	0	10.610/−3
	*LRSAM1* (F) [93,94]	9:130253524	Non-synon	p.L484M	Ctg/Atg	Missense	18, 23	0.796	0.010	0.935	0	0.787/−1
	*CYFIP1* (A) [95]	15:22960872	Non-synon	p.D716N	Gac/Aac	Missense	28, 30	0.767	0.009	0.999	0	0.067/1
**HI2215 60%:40%**	*FPGT- TNNI3K* (F) [96]	1:74836022	Non-synon	p.G673V	gGc/gTc	Missense	34, 53	0.802	0.009	0.998	0	0.787/−3
	*MAP1A* (P) [96]	15:43820377	Non-synon	p.P2235S	Ccc/Tcc	Missense	65, 67	0.793	0	0.998	0	0.787/−1
	*MAP1A* (P) [96]	15:43819396	Non-synon	p.Y1908H	Tac/Cac	Missense	13, 21	0.782	0.004	0.994	0	0.787/2
**HI2244 32%:68%**	*KDM5B* (P) [97,98,99]	1:202724501	Non-synon	p.Y320K	aTa/aAa	Missense	52, 47	0.808	0.007	0.988	0	0.787/−4
	*TRPM2* (P) [97,98,99]	21: 45826549	Non-synon	p.I954F	Atc/Ttc	Missense	17, 16	0.786	0.008	0.997	0	12.615/−3
	*RAI1* (A) [100]	17:17699284	Non-synon	p.P959A	Ccc/Gcc	Missense	37, 26	0.763	0	0.992	0	0.787/−1
**HI2278 8%:92%**	*DCHS1* (F) [39]	11:6662745	Codon Change Plus Codon Insertion	p.L32LW	Ctg/ctCTGg	None	12, 11	0.700	0	0.979	0.02	0/0
**HI2843 25%:75%**	*UBE2E2* (P) [101]	3:23631311	Non-synon	p.Y198H	Tac/Cac	Missense	64, 33	0.787	0	0.994	0	0.787/2
	*PCDHB15* (P) [45]	5:140625618	Non-synon	p.R157W	Cgg/Tgg	Missense	33, 61	0.754	0.007	0.942	0	24.449/−3
	*SCG2* (F) [102]	2:224463227	Non-synon	p.E257D	gaG/gaC	Missense	70, 64	0.750	0	0.993	0.01	0.787/2
**HI2879 18%:82%**	*TRIM9* (F) [103]	14:51448554	Non-synon	p.R623Q	cGg/cAg	Missense	64, 69	0.774	0	1	0	0.787/1
	*SOX7* (A) [104]	8:10583274	Non-synon	p.Y432H	Tac/Cac	Missense	25, 33	0.766	0	0.997	0	0.787/2
	*ANKFN1* (P) [105]	17:54559849	Non-synon	p.G744R	Ggg/Agg	Missense	28, 16	0.715	0	0.995	0.04	26.000/−2
	*DCHS1* (F) [39]	11:6662745	Codon Change Plus Codon Insertion	p.L32LW	ctg/ctCTGg	None	11, 12	0.700	0	0.979	0.02	0/0
**HI2898 85%:15%**	*MPHOSPH8* (F) [106]	13:20242552	Non-synon	p.Y736C	tAc/tGc	Missense	39, 28	0.779	0.007	0.999	0	0.787/−2
	*GABRQ* (A) [107]	X:151819020	Non-synon	p.S292F	tCc/tTc	Missense	9, 16	0.749	0.008	0.963	0	0.787/−2

ID Number represents the AGRE identifier. For the 30 females with ASD in this table, the average age ± standard deviation was 7.7 ± 2.6 years and age range was 5–16 years. Final selection criteria: Classification Score > 0.7; GERP2 < 0.01; PolyPhen2 > 0.9; SIFT < 0.03. * (A) = Known clinically relevant gene for autism; (F) = Neurodevelopment functional gene for autism; (P) = Known paralogue of an autism gene; NI = not informative; XCI = X chromosome Inactivation (%); ? indicates an unspecified amino acid variation; [ ] represents literature citations for each gene description.

**Table 2:** Summary of Putative Disease Causing Gene Variants Identified by Exome Sequencing.

Gene Variant Category	Number of Subjects (%)	Number of Genes (%)	Range
Known ASD Genes	11 (37%)	12 (15%)	0–2
Paralogues of Known ASD Genes	16 (53%)	23 (30%)	0–2
Neurodevelopmental Function Genes	23 (77%)	43 (54%)	0–7

Six females (HI0605, HI2898, HI1739, HI2172, HI2244 and HI2879) possessed a single variant of a known candidate ASD gene meeting our final selection criteria for pathogenicity in addition to one or more paralogues of ASD genes or gene variants with functional influence over cell growth and neurodevelopment. Fourteen females (HI0714, HI0751, HI0765, HI0855, HI0868, HI1157, HI1228, HI1305, HI1375, HI1422, HI1433, HI1954, HI2215 and HI2843) possessed 2 to 4 variants of ASD gene paralogues or variants with functional roles of influence over ASD but without a conclusive or definable causal mutation. Variants of multiple putative candidate genes for ASD were identified for Subjects HI0405, HI0558 and HI0793.

Using XCI data, we found that eight of the 30 females had high skewness (XCI > 80%:20%) and an additional six females showed moderate skewness (XCI 65%:35% to 80%:20%). Two of the eight females with high skewness and two females with moderate skewness possessed an X-linked gene variant meeting the final selection criteria. When considering the 78 total genes identified meeting our final selection criteria, X-linked gene variants were disproportionally more likely to be found among the females with autism spectrum disorder who exhibited moderate to high level XCI skewness than females without XCI skewness (*OR* = 6.4, 95% *CI*: 0.68, 60.5, *p* = 0.1) but this relationship did not achieve statistical significance. Similarly, females who exhibited XCI skewness were more likely to possess an X-linked gene variant than females without XCI skewness, (*OR* = 6.0, 95% *CI*: 0.58, 61.8, *p* = 0.132).

## 3. Discussion

We chose females with ASD to study from the age of 5 to 16 years from multiplex families with more than one affected family member in order to increase the likelihood of identifying a single gene as a causative factor using a descriptive case study approach. The females were selected from the AGRE, a biorepository of specimens and clinical data from well-characterized families with children with ASD enrolled for genetic research studies. Females with ASD from multiplex families were also examined to determine whether an overabundance of X-linked genes could be identified contributing to ASD by using whole exome sequencing and XCI assays and if X-linked genes were more frequently disturbed in those females with XCI skewness.

### 3.1. Single Gene Variants

Our investigation identified four females (HI0555, HI0890, HI1402 and HI2126) with single gene variants of clinically relevant candidate or known genes for ASD with a high likelihood of causality. Subject HI0555 possessed a non-synonymous, missense mutation of the *SETD2* gene located on chromosome 3 with high likelihood to have a deleterious effect on gene expression and/or function. The *SETD2* encodes a Huntingtin-interacting protein B related to Huntington disease and known expression in the brain [27,28]. The *SETD2* gene is known as an autism susceptibility gene reported in autism [12] and was the highest rated and most likely candidate gene in this case. Subject HI0890 possessed a non-synonymous, missense mutation of the *BTAF1* gene located on chromosome 10 which is implicated as a helicase and sequence-specific binding transcription factor activity [63]. Subject HI2126 possessed a non-synonymous, missense mutation in a known candidate gene for ASD, *ANKRD11* which is located on chromosome 16 and likely to be causative in this case. The *ANKRD11* gene encodes an ankryin repeat domain-containing protein which inhibits ligand-dependent activation of transcription with mutations causing the KBG syndrome characterized by craniofacial features, short stature, skeletal anomalies, seizures and intellectual disability [90,91]. No physical examination or cognitive data were available from AGRE on this 12 year old female diagnosed with autism.

### 3.2. Involvement of Skewness of X Chromosome Inactivation and Putative Disease Causing Genes

High XCI skewness (>80%:20%) was observed for eight of our females with autism and two of these females possessed putative variants of X-linked genes (HI1402 and HI2898) which may contribute to the phenotype. Skewing of X-chromosome inactivation may increase expression of recessive disease causing variants on the X-chromosome. Subject HI1402 with an XCI status of 85%:15% indicating high skewness also possessed a non-synonymous, missense mutation of an X-linked gene (*IL1RAPL1*) which is a recognized candidate gene for ASD [12]. *IL1RAPL1* had the highest criteria selection score of the four genes meeting initial selection criteria and the most likely cause of ASD in this female. The *IL1RAPL1* gene encodes a protein with the highest expression in brain neurons and participates in the regulation of neurite outgrowth via interaction with neuronal calcium sensors thereby regulating synaptic formation and modulation of synaptic transmission [74].

Subject HI2898 with an XCI status of 85%:15% showing high skewness possessed a variant of the X-linked gene (*GABRQ*) found to be in the top two genes in the selection process. The *GABRQ* (gamma-aminobutyric acid A receptor theta) gene encodes a receptor protein for GABAergic neurotransmission in the mammalian central nervous system [107]. GABA is a major inhibitory neurotransmitter. *GABRQ* expression is distributed in the amygdala, hippocampus, anterior hypothalamus and cortex [107]. Subject HI2278 with an XCI status of 8%:92% possessed an X-linked gene (*PAK3*) in the top 25 genes but was excluded due to strict filtering requirements (*i.e.*, PolyPhen 2) and a correct classification score of >0.50. However, the *DCHS1* gene was identified which encodes a transmembrane cell adhesion molecule that belongs to the protocadherin superfamily and acts as a ligand for FAT4, another protocadherin protein. Both DCHS1 and FAT4 form an apically located adhesive complex in the developing brain [39].

The five remaining females (HI0751, HI1143, HI1375, HI1433 and HI2879) with high XCI skewness ranging from 93%:7% to 82%:18% showed no X-linked gene variants meeting the final selection criteria for pathogenicity. Other variations of possible clinical relevance in autosomal chromosomes were observed for these subjects. Subject HI1433 with an XCI status of 93%:7% indicating extreme skewness possessed a variant of the *FAT2* gene located on chromosome 5 which is a member of the cadherin-related FAT tumor suppressor homolog 2 (Drosophila) [77,78,79]. Subject HI2879 with an XCI status of 18%:82% showing high skewness possessed a variant of the *SOX7* (SRY-Box 7) gene which encodes a SOX protein, acting as a transcription factor regulating diverse developmental processes [104]. Based upon our investigation, XCI skewness in these females did not contribute to the presentation of autism.

Moderate skewness (65%:35% to 80%:20%) was observed for six females (HI0765, HI1305, HI1739, HI1954, HI2244 and HI2843). Two of these females possessed putative variants of X-linked genes that may have contributed to their phenotype. Subject HI1305 with an XCI status of 73%:27% possessed a variant for *PIR*, a highly conserved X-linked iron-binding nuclear protein gene which functions as a transcriptional co-regulator and contributes to regulation of cellular processes and may promote apoptosis when over expressed [70]. An associated disorder for this gene when disturbed is extratemporal epilepsy. Three genes were identified for Subject HI0765 with an XCI status of 77%:23% with moderate skewness including *GPRASP2* located on the X chromosome which encodes a protein that may regulate a variety of G-protein coupled receptors associated with autism spectrum disorders and schizophrenia [48], as an X-linked gene approaching high skewness becomes an important candidate for causation of ASD in this female. The *KCNC2* and *ASPM* genes were the highest rated and became likely candidates

### 3.3. Other Autosomal Putative Disease Causing Genes

The cadherin, protocadherin and ankyrin repeat gene families were the most commonly altered putative disease causing gene variants identified in our study. Subject HI0558 possessed a variant of *PCDH8*, a member of the cadherin superfamily of genes and functions in cell adhesion in a CNS-specific manner possibly playing a role in activity-induced synaptic reorganization underlying memory with down-regulation of dendritic spines, primarily in the hippocampal area [35]. In addition this affected female possessed a variant of the *CTNNA3* gene which is located on chromosome 10 and encodes an alpha-t-catenin which plays a role in functional cadherin-mediated cell adhesion. A possible association of this gene in Alzheimer disease has been proposed [36]. Subject HI0855 possessed a variant of *CDH6* located on chromosome 5 encoding a known cadherin playing a role in cell-cell adhesion and implicated in autism [60]. Subject HI0605 possessed a non-synonymous, missense mutation of the *CCDC64* (coiled-coil domain containing, 64), a recognized ASD gene on chromosome 12 [12,38]. CCDC64 is a component of the secretory vesicle machinery in developing neurons that acts as a regulator of neurite outgrowth in the early phase of neuronal differentiation which when disturbed is associated with neuronitis [38]. Subject HI1739 possessed a non-synonymous missense mutation of the *ASB3* gene located on chromosome 2 and related to the ankryin repeat gene family known to play a role in brain development and function including autism [88]. *GRM4* (glutamate receptor, metabotropic, 4) is a known ASD gene involved with glutamate, a major excitatory neurotransmitter in the central nervous system [86]. Subject HI2172 possessed a non-synonymous, missense mutation of the *CYFIP1* (cytoplasmic FMRP-interacting protein 1) gene located on chromosome 15 which encodes a protein that interacts with the familial mental retardation protein (FMRP) that when disturbed, causes the fragile X syndrome by impacting on development and maintenance of neuronal structures [108,109]. Individuals with the 15q11.2 BP1-BP2 microdeletion or Burnside-Butler syndrome are known to have developmental and speech delay involving the *CYFIP1* gene with an increased rate of aberrant behavior and autism [95].

## 4. Experimental Section

### 4.1. Samples from Females with Autism

Thirty Caucasian females (average age, 7.7 ± 2.6 years; age range, 5 to 16 years) were selected with confirmed diagnosis of autism. We chose to undertake whole exome sequencing from well-characterized females with autism with a positive family history (e.g., affected brothers) classified as multiplex families with autism and having a high probability of causation due to gene disturbances. We selected affected females from the Autism Genetic Research Exchange (AGRE) repository (www.agre.autismspeaks.org) for a descriptive next generation whole exome sequencing case study. The family members with autism in the AGRE were recruited and enrolled after screening and diagnostic assessments were performed with autism-related testing instruments (e.g., ADOS). Medical examinations and neurology evaluations were undertaken to collect family, pregnancy and medical history data along with physical and anthropometric measures and neurological recordings. Blood was also collected at AGRE for lymphoblastoid cell line development, plasma storage and DNA isolation. Normal chromosome analysis (karyotype) and fragile X syndrome DNA testing were completed previously in all female subjects selected from AGRE. DNA from parents or other family members were not analyzed as a component of our study.

### 4.2. Whole Exome Sequencing

#### 4.2.1. Exome Sequencing Methods

Genomic DNA (5 µg) samples were sent to the Silicon Valley Biosystems, Foster City, CA, USA for whole exome sequencing via paired-end next generation sequencing (NGS) approach using standard protocols with the Illumina HiSeq2000 platform (http://www.service.Illumina.com) and Agilent SureSelect Human All Exon v4–51Mb (http://www.genomics.agilent.com). The primary sequence raw data produced by the sequencing phase of our study was aligned to reference, the variants called and functional significance of each variant determined and rank-ordered into a list of functional variants (in order of decreasing pathology) that were correlated with or causative for the autism phenotype. The raw data was in the form of *fastq* files, containing the sequence reads and quality scores of the NGS sequencing runs. The individual reads were initially aligned by mapping to the reference genome using Burrows-Wheeler aligner (BWA), a software program for mapping low-divergent genomic sequences of up to 100 base pairs against a large reference genome (http://www.bio-bwa.sourceforge.net). Variant calling procedures then identified genomic sequence variants from the NGS sequencing runs or the absence of variants in the sample of interest relative to a reference sequence. The platform used to produce variant calls was an amalgamation of tools and methods uniquely combined and specifically calibrated for enhanced performance incorporating *Genome Analysis Toolkit* (*GATK*) V2.39 (https://www.broadinstitute.org/gatk/). A number of intermediate steps were applied to improve the quality/accuracy of the alignment and variant calling results prior to the creation of the final list of variants. Sequence reads that were likely to represent duplicates were marked to be ignored by the downstream variant-calling tools. Additionally, realignment around known insertions and deletions was performed, and quality scores recalibrated based upon the number of reads. The list of variants was produced in the form of a .vcf file annotated to reflect the results of quality control filters. This established and calibrated alignment and variant calling platform performs well and can reliably predict calls for single insertion and deletion events (up to 50 bp with between 97% and 99.9% sensitivity and specificity). The average number of reads per generated fragment was 64 for each of the 30 females with autism. 

#### 4.2.2. Data Analysis

The resulting .vcf file contained annotated variants with scientific features, including sequence, gene-, variant-, and transcript-level annotations. Annotations include characteristics of the observed variant such as the genomic coordinates or the variant’s genomic region (e.g., exonic, upstream *etc.*), predicted effects on the various transcripts (e.g., missense, frame-shift, *etc.*), observed frequencies of the variant in control populations and affected individuals, curated knowledge from both proprietary and public databases, and pathogenicity predictions (e.g., Polyphen and SIFT). The machine learning tool utilized both the 2012 April 1K Genome release reflecting the frequency at the position and variant levels and the European Exome Sequencing Project (ESP6500) (https://esp.gs.washington.edu/drupal/) at the variant level which reflects the frequency of variants as observed in the ESP6500 dataset of healthy individuals in generating the correct classification score. The regulatory significance of variants were derived from ENCODE data. A classification engine developed by Silicon Valley Biosystems was used to create a pathogenicity score based on a machine learning technique approach. The model is trained and validated using 150,000 gene polymorphisms and mutations from a large set of variants with known pathogenicity. The classification accuracy exceeds 99% and thus the score provides the probability that a particular variant has an effect on gene expression or protein structure. The classifier was trained using known disease-causing mutations and known benign polymorphisms, each of which was annotated with attributes such as location relative to splice junctions, type of non-synonymous amino acid substitution and allele frequency in the population. The classifier was validated on a similarly sized set of variants and was shown to perform with 93% sensitive and 97% specificity. Prioritization of putative functionally relevant variants based on the classification score was performed and these variants were additionally classified with other means of determining pathogenicity such as SIFT, PolyPhen2, Blosum and Blosum62. The variants that were the most highly predicted to be functionally significant were examined across the study cohort for clustering within and around genes hypothesized as relevant to the phenotype.

#### 4.2.3. Gene Filtration/Selection Parameters

A high filter system was utilized whereby the indels and quality disqualified (QD) parameters were identified and removed based on the bioinformatics outlined in our study approach. The classification score (range 0 to 1) was generated as our primary delimiting factor in identifying novel or candidate genes for autism in the affected females. The classifier uses machine learning algorithms to predict a variant’s functional impact on a gene with higher scores for genomic variants considered more pathogenic or disease-causing changes at the gene level. Thus, we applied an initial classification cutoff of >0.5 with final selection criteria >0.7. Genomic Evolutionary Rate Profiling (GERP2), an indicator of the prevalence of the genomic variants in the general population, was assigned a cutoff value of <0.01 and remained at <0.01 as final selection criteria. GERP2 identifies constrained elements in multiple alignments by quantifying substitution deficits. These deficits represented substitutions that would have occurred if the element was neutral DNA, but did not occur because the element was under functional constraint. The initial Polymorphism Phenotyping v2 (PolyPhen2) cutoff value was >0.85 with the final selection criteria >0.9 and the initial Sorts Intolerant From Tolerant (SIFT) cutoff valuewas <0.05 with a final selection criteria <0.03 were predictors of pathogenicity or disease causing changes. The Blosum (BLOcks SUbstitution Matrix) score reports the ratio between the frequency of an amino acid substitution in the Human Gene Mutation Database (HGMD) to the frequency of this amino acid substitution in known polymorphism data sets. Numbers greater than 1 indicate this variant was observed more in diseased individuals than in controls and less likely to be tolerated. We also used the Blosum62 matrix to score alignments between evolutionarily divergent protein sequences based on local alignments. Blosum62 is a log-odds score for each possible substitution of the 20 standard amino acids. Higher Blosum62 numbers represent more closely related species comparisons and provide a general indication of frequency of the substitution. Negative numbers indicate less frequent substitutions and a lower likelihood to be tolerated (e.g., negative values = unexpected or rare substitutions).

The list of disease causing genes were developed using the primary selection criteria (*i.e.*, classification score) and then adding cutoff levels for other predictive parameters (GERP2, PolyPhen2, and SIFT). The initial list of genes and genomic variants used the classification score >0.5 alone and then the cutoff levels of the other predictive parameters (GERP2, PolyPhen2 and SIFT) were included. The classification score was then raised to >0.7 to generate the final list of genes. These putative candidate genes and genomic variants were then subjected to further screening for biological significance for neural development, function and known neurological disorders using Online Mendelian Inheritance in Man (OMIM, http://www.ncbi.nlm.nih.gov/omim), GeneCards (http://www.genecards.org/), PubMed and other online websites and databases.

#### 4.2.4. Clinical Relevance to ASD

Following the recommendations of the American College of Medical Genetics and Genomics (ACMGG) in reporting clinical exome and genome sequencing data, we analyzed only the primary findings, in depth, which is termed as the pathogenic alteration in a gene or genes relevant to the diagnostic indication for sequencing study, *i.e.*, causation of autism in females. Only clinically relevant candidate or known existing genes for autism were included for extended analysis. Incidental findings are those pathogenic or likely pathogenic alterations in genes that are disease associated or causing but not apparently relevant to a diagnostic indication for which sequencing was undertaken (*i.e.*, in our study on autism [12]) and were not further investigated in our study. We did not include mutations of recognized autosomal recessive genes causing human disorder (e.g., *GJB2* or *GJB6*, gene mutations causing hearing loss). It is estimated that about 1% of sequencing reports would include an incidental variant.

### 4.3. X Chromosome Inactivation in Females with Autism

Genomic DNA isolated from blood was used as a template for polymerase chain reaction (PCR) amplification, to identify the CAG polymorphic region of the *AR* gene. Prior to the PCR amplification, 200 ng of genomic DNA was digested with the methyl-sensitive restriction enzyme *Hpa*II [21]. Approximately 50 ng of digested or undigested genomic DNA was used as a template for PCR amplification to determine the peak height of the polymorphic PCR fragment of the *AR* gene using the following primers: forward 5' TCCAGAATCTGTTCCAGAGCGTGC 3' and reverse 5' GCTGTGAAGGTTGCTGTTCCTCAT 3' with the forward primer fluorescently labeled with 6-FAM. The lengths and peak heights of the resulting PCR fragments are determined with the use of capillary electrophoresis and fragment separation software with the ABI 3100 DNA sequencer (Applied Biosystems, Carlsbad, CA, USA) using established protocols [18,22].

The digestion process preferentially degrades activated (unmethylated) over inactivated (methylated) DNA. Undigested DNA is preferentially amplified and produces larger peak heights. The peak height values for digested DNA is normalized using peak height values for the undigested DNA for each subject. The percentage of XCI for each *AR* allele was then calculated using the following formula: (d1/u1)/[(d1/u1) + (d2/u2)]; d1 = peak height of digested DNA from the first allele and u1 = peak height of undigested DNA from the first allele; d2 = peak height of digested DNA from the second allele and u2 = peak height of undigested DNA from the second allele. Highly skewed XCI is defined as >80% calculated ratio for either one of the *AR* gene alleles in the digested DNA sample. To ensure the reproducibility of XCI results and equal amplification of both alleles, the digestion, PCR amplification, and genotyping were repeated up to three times in several samples.

XCI status for each subject was assigned to one of the three mutually exclusive categories: (i) randomly selected inactivation of either allele from each X chromosome (XCI = 50%:50% to 64%:36%); (ii) moderately skewed inactivation favoring 1 allele of one of the X chromosomes (XCI = 65%:35% to 80%:20%); and (iii) highly skewed inactivation of a single allele representing one X chromosome (XCI > 80%:20%). The relative frequency of random, moderate, and highly skewed XCI categories was determined for the females with autism. The binomial frequency distribution of X-linked gene variants (present or absent) among females with autism spectrum disorder exhibiting moderate and high levels of XCI skewness were approximated using Yates’ chi-squire test and odds ratios were calculated with 95% confidence intervals.

The top genes chosen were based on the final selection criteria identified for each female and evaluated for biologic function and whether each gene was previously reported as a clinically relevant candidate or known gene for autism [12]. The X chromosome inactivation data and XCI status on each female were used to support whether an X-linked gene played a role in the causation of autism in the affected female if XCI skewness was found.

## 5. Conclusions

In summary, we shared our experience using a descriptive next generation whole exome sequencing case study approach examining 30 well-characterized Caucasian females with autism between 5 and 16 years of age recruited from multiplex families enrolled at the AGRE for research purposes. Interpretation of DNA findings are limited due to an inability to study other affected and non-affected family members to determine whether the gene variants were de-novo or inherited in origin for correlation with clinical phenotypes and lack of Sanger sequencing confirmation. Using strict selection criteria, four females (13%) were found to possess single gene variants of known candidate genes for ASD with a high likelihood of causality. We also identified multiple plausible candidate genes [some known (e.g., *CCDC64* in subject HI0605) and some novel (e.g., *CHAC1* in subject HI0855)] in 77% of the remaining females. In most females, we found more than one gene that could contribute to the causation of autism. This compares with a recent report of molecular findings among 2000 consecutive patients (primarily pediatric age) referred for clinical whole exome sequencing for evaluation of suspected genetic disorders at a large USA medical center in which 25% received a molecular diagnosis. About 60% of the diagnostic mutations were not previously reported [110]. By phenotypic category, 36% of those presenting with neurological involvement were found to have a molecular diagnosis while 20% of those from the non-neurological group had a diagnosis. We not only performed whole exome sequencing in our females with autism but also performed X chromosome inactivation (XCI) studies for evidence of X-linked genes in the females showing non-random XCI skewness. Moderate and high XCI skewness was seen in 14 of the 30 females and those with skewness were 4 times more likely to show a gene variant on the X chromosome.
